# Impact of a research-action on vaccination indicators in the state of Minas Gerais, Brazil

**DOI:** 10.11606/s1518-8787.2024058005484

**Published:** 2024-03-04

**Authors:** Janaina Fonseca Almeida Souza, Thales Philipe Rodrigues da Silva, Thais Moreira Oliveira, Aline Mendes Vimieiro, Antônia Maria da Silva Teixeira, Adriana Coelho Soares, Elice Eliane Nobre Ribeiro, Giselle Lima de Freitas, Eduarda Dantas Gaspar, Fernanda Penido Matozinhos

**Affiliations:** I Universidade Federal de Minas Gerais Escolas de Enfermagem Programa de Pós-graduação em Saúde e Enfermagem Belo Horizonte MG Brasil Universidade Federal de Minas Gerais. Escolas de Enfermagem. Programa de Pós-graduação em Saúde e Enfermagem. Belo Horizonte, MG, Brasil; II Secretaria de Estado da Saúde de Minas Gerais Belo Horizonte MG Brasil Secretaria de Estado da Saúde de Minas Gerais, Belo Horizonte, MG, Brasil; III Universidade Federal de São Paulo Escola Paulista de Enfermagem Departamento de Enfermagem na Saúde da Mulher São Paulo SP Brasil Universidade Federal de São Paulo. Escola Paulista de Enfermagem. Departamento de Enfermagem na Saúde da Mulher. São Paulo, SP, Brasil; IV Santa Casa de Belo Horizonte Belo Horizonte MG Brasil Santa Casa de Belo Horizonte. Belo Horizonte, MG, Brasil; V Sociedade Brasileira de Imunizações São Paulo SP Brasil Sociedade Brasileira de Imunizações. São Paulo, SP, Brasil; VI Universidade Federal de Minas Gerais Escola de Enfermagem Departamento de Enfermagem Materno-Infantil e Saúde Pública Belo Horizonte MG Brasil Universidade Federal de Minas Gerais. Escola de Enfermagem. Departamento de Enfermagem Materno-Infantil e Saúde Pública. Belo Horizonte, MG, Brasil

**Keywords:** Vaccination Coverage, Risk Management, Child, Health Impact Assessment

## Abstract

**OBJECTIVE:**

Analyze the impact of the state research-action project on immunization indicators (vaccination coverage – VC, homogeneity of vaccination coverage – HVC, dropout rate – DR, and risk rating) before and after the intervention in municipalities and priority Regional Health Administrations/Regional Health Superintendencies (RHA/RHS).

**METHODS:**

The state research-action project was a before-after community clinical trial conducted in 212 municipalities belonging to eight RHA/RHS in the state of Minas Gerais, Brazil. The study sample comprised RHA/RHS with a decreasing trend for routine vaccination coverage in children under one year from 2015 to 2020. This study used secondary VC and DR data from 10 immunobiologicals recommended for children younger than two years from January to December 2021 (pre-intervention period, prior to the state research-action project) and from January to December 2022 (post-intervention period). The categorical variables were presented in proportions, and initially, a comparison was made between those of DR, HVC, and the risk rating for the transmission of vaccine-preventable diseases, according to the two periods (2021 and 2022), using the McNemar test.

**RESULTS:**

All immunization indicators increased after conducting the research-action project. In 2021, 80.66% of the state’s municipalities had a risk rating for the transmission of vaccine-preventable diseases as “high and very high.” In 2022, the value reduced to 68.40%.

**CONCLUSIONS:**

Risk rating for the transmission of vaccine-preventable diseases is an important mechanism to assist managers in defining priorities. The state research-action project used a method that enabled the construction and execution of unique action plans for each municipality, directing the improvement of immunization indicators in the state.

## INTRODUCTION

The *Programa Nacional de Imunização* (PNI – Brazilian National Immunization Program), created in 1973, is coordinated by the Ministry of Health, with co-participation from state and municipal health departments^1,2^. It is one of the most recognized and complete immunization programs in the world^1,2^, primarily due to the collective and individual strategies used, which guarantee high vaccination coverage over the years for the majority of immunobiologicals offered free of charge to the population^3,4^.

Over the years, the PNI has significantly reduced morbidity and mortality from vaccine-preventable diseases in the Brazilian population, constituting an efficient public health policy^2^. In this sense, vaccination actions are considered one of the most successful, cost-effective health interventions^5,6^ in all life cycles (children, adults, pregnant women, and older people) and specific populations (such as Indigenous peoples)^1,3^.

However, a sharp reduction in vaccination coverage rates has been observed in Brazilian territory since 2016^7^, exacerbated by the COVID-19 pandemic^11,12^. This scenario highlights a serious problem for collective immunity and puts the population at risk of transmitting vaccine-preventable diseases^13^.

In Minas Gerais—the fourth state with the largest territorial area and the second in number of inhabitants, located in the Southeast region of the country^14^— the drop in vaccination coverage and the increase in the number of municipalities at high risk for the transmission of vaccine-preventable diseases followed a similar trend compared to the other Brazilian states^10^ from 2015 to 2020. Considering the 28 Regional Health Administrations/Regional Health Superintendencies (RHA/RHS) in the state of Minas Gerais as the unit of analysis, the lowest proportion of RHA/RHS that achieved the recommended vaccination coverage targets for the immunobiologicals studied in 2020 was identified^11^.

The drop in vaccination coverage is multifactorial^1^. In addition to situational diagnosis, overcoming the challenges of low coverage requires integrating the different areas of the health sector, the social sectors, and education, identifying pockets of susceptible individuals, and developing strategies to maintain high vaccination coverage^1,9^.

From this perspective, and considering the vulnerable situation of children, in 2021, the Vaccination Studies and Research Center of the School of Nursing of the Federal University of Minas Gerais (NUPESV-EEUFMG) coordinated with the Secretary of State for Health of Minas Gerais (SES-MG) in a research-action project entitled “*Estratégias para o Aumento de Coberturas Vacinais nas Crianças Menores de Dois Anos no Estado de Minas Gerais, Brasil: uma Pesquisa-Ação*,” aiming to improve vaccination coverage of children through workshops and construction of action plans unique to the reality of each municipality in the state.

The research-action (RA) refers to a methodology applied in various scientific subjects, also covering the health area, due to its ability to deeply understand an object of study and, at the same time, improve the practice associated with it^15^. The technique of carrying out workshops was chosen to understand the weaknesses and potential of each municipality concerning the immunization of children under two years of age and to promote a space for the exchange of ideas and the collective construction of knowledge. In this methodology, research participants are known to have the opportunity to influence and modify reality^15^. Workshops offer the chance for negotiation, argumentative debate, and dialogue between participants, thus becoming a powerful tool to engage and transform reality as they facilitate the integration of theoretical and methodological issues and ethical and political implications^16^.

The objective of this study is to analyze the impact of the state action research project on vaccination coverage (VC), especially on the homogeneity of vaccine coverage indicator (HVC), dropout rate (DR), and risk rating for transmission of vaccine-preventable diseases in the municipalities of Minas Gerais, Brazil, comparing the years 2022 and 2021.

## METHODS

The study sample comprised the RHS/RHA with a decreasing trend for routine vaccination coverage in children under one year of age from 2015 to 2020^10^, namely, RHS Alfenas: 24 municipalities, RHS Barbacena: 31 municipalities, RHS Coronel Fabriciano: 35 municipalities, RHS Governador Valadares: 51 municipalities, RHA Ituiutaba: nine municipalities, RHA Leopoldina: 15 municipalities, RHS Passos: 27 municipalities, and RHA São João Del Rey: 20 municipalities, totaling 212 municipalities, i.e., 24.85% of the state’s total. The RHS/RHA that made up the sample of this study were selected based on the previous study conducted by Souza et al.^10^ (2022), which identified a decreasing trend for immunobiologicals recommended for children under two years of age in at least five of these regional administrations.

The workshops lasted 12 hours, they were held in different contexts and were led by professionals from SES-MG and NUPESV-EEUFMG, requiring detailed prior organization. Cooperation between both parties was fundamental, from creating the research project to the ongoing collaboration. Workshops were held with primary healthcare professionals (especially nurses), nursing technicians, and assistants who work directly with vaccination in municipalities, managers, coordinators of epidemiological surveillance, and primary healthcare and health secretaries, in addition to other external partners (representatives of universities, Municipal Health Councils, Council of Municipal Health Secretaries) to operationalize the project. The workshops had the following steps: 1. Dialogued presentation: analysis of vaccination coverage in children under two years of age in municipalities and in RHA/RHS, conversation and brainstorming with triggering questions for problematization; 2. Start of group activities and construction of Municipal Action Plans; 3. Presentation, through a rapporteur from each group, of the discussions held to begin the construction of the action plans.

After the workshops, each participating municipality had 15 days to send its action plan to the respective RHA/RHS after agreement with managers, technical team, and care team and approval by the Municipal Health Council. Consideration was given to the following strategic axes to prepare the plans: people management, social communication, strategic partnerships, infrastructure and logistics, management coordination, and monitoring and evaluation. The workshops took place between March and June 2022, with 515 participants, including health managers, health surveillance coordinators, and external partners. Each municipality had, on average, four representatives in the workshops, led by a coordinating team.

This study used secondary VC and DR data from ten immunobiologicals recommended for children under two years of age in 2021 (pre-intervention period, prior to the state research-action project) and 2022 (post-intervention period).

The immunobiologicals recommended for children under 2 years of age, in both years (2021 and 2022), were: oral vaccine against rotavirus (2^nd^ dose of rotavirus vaccine in the Unified Health System (SUS) plus the 2^nd^ dose of rota-pentavalent in the private network), meningococcal C disease vaccine (2^nd^ dose of meningococcal C and 2^nd^ dose of meningococcal ACWY), pneumococcal disease vaccine (2^nd^ dose of pneumococcal 10V and 2^nd^ dose of pneumococcal 13V), pentavalent vaccine (3^rd^ dose of pentavalent vaccine plus 3^rd^ dose of hexavalent vaccine in the private network), polio vaccine (3^rd^ dose of VIP, VOP, pentavalent in the private network and hexavalent also in the private network), yellow fever vaccine (single dose, initial dose, and 1^st^ dose), 1^st^ dose of the triple viral vaccine (1^st^ dose of the triple viral, 1^st^ dose of the quadruple viral, and 1^st^ dose of the tetra viral), 2^nd^ dose of the triple viral vaccine (2^nd^ dose of the triple viral, 2^nd^ dose of the quadruple viral, 2^nd^ dose, and single dose of tetra viral), hepatitis A vaccine (1^st^ dose considered), and chickenpox vaccine (1^st^ dose of chickenpox and 1^st^ dose of tetra viral).

All information was extracted from the *Sistema de Informações do Programa Nacional de Imunizações* (SIPNI – National Immunization Program Information System), available at <sipni.datasus.gov.br>. The BCG and Hepatitis B vaccines were not evaluated, as these are primarily administered in maternity wards, which could cause a bias in the analyses.

For the 2021 analyses, the VC was calculated using as the denominator the population of the Live Birth Information System (SINASC) under one year old in 2019. For 2022, the SINASC for 2020 was used, always considering the most updated information. The doses (immunizing dose or the dose that completes the vaccination schedule) applied by age group and immunobiological, according to the National Vaccination Calendar of the Ministry of Health, were used in the numerator.

VC rates were categorized according to the targets established by the PNI (greater than or equal to 90% for the oral human rotavirus vaccine and greater than or equal to 95% for other immunobiologicals) as “very low” (< 50%), “low” (≥ 50% and less than the target), and “adequate” (≥ the target)^13^.

The homogeneity of vaccination coverage (HVC) between the vaccines analyzed was also verified, following the definition adopted by a previous study by Braz et al*.*^13^, agreed by the Unified Health System (SUS), through the Organizational Contract for Public Health Action (COAP): “adequate” when HVC levels were ≥ 75% to ≤ 100% for the ten vaccines with adequate coverage (≥ the target), “low” when they were ≥ 50% to < 75%, and “very low” when the percentage was < 50% for the ten vaccines analyzed.

The DR was also calculated for multidose vaccines, namely, vaccine against meningococcal disease C (for 2022), pentavalent vaccine (for 2021 and 2022), pneumococcal disease vaccine (for 2021 and 2022), polio vaccine (for 2021 and 2022), and oral human rotavirus vaccine (for 2021 and 2022), classified as “low” DR (< 5%), “medium” DR (≥ 5% to < 10%), and “high” DR (≥ 10%)^13^. The difference between the number of first and last doses of the vaccination schedule was considered to calculate the DR, divided by the number of first doses applied, multiplying the result by 100^13^.

The municipalities were categorized based on their population sizes, as previously defined in the study by Braz et al.^13^ This classification considers three groups: small municipalities, which have a population equal to or less than 20 thousand inhabitants; medium-sized municipalities, with populations ranging between 20,001 and 100 thousand inhabitants, and large municipalities, with a population equal to or greater than 100,001 inhabitants.

Finally, the municipalities participating in the research were classified according to the risk of transmission of vaccine-preventable diseases into five strata, according to Braz et al.^13^, for the two years of analysis:

•“Very low”: municipalities with HVC = 100%;
•“Low”: municipalities with HVC from ≥ 75% to < 100%, with adequate VC for polio and triple viral (international commitment to eliminate diseases) vaccines, and also the pentavalent vaccine, considered a “standard marker” of quality vaccination service (schedule of three injectable doses);
•“Medium”: municipalities with HVC ≥ 75% and < 100% and VC below the target for one or more of the polio, triple viral, or pentavalent vaccines;
•“High”: municipalities with HVC < 75%, regardless of vaccination coverage;
•“Very high”: municipalities with HVC < 75%, high DR (≥ 10%) for any of the vaccines evaluated and with large population size, and municipalities without vaccination records for any vaccine, regardless of population size.

Due to the small number of municipalities classified as “medium” and “very high” risk for the transmission of vaccine-preventable diseases, these categories were grouped into “low and medium” and “high and very high” risk of transmission of vaccine-preventable diseases.

The categorical variables were presented in proportions, and initially, the values of DR, HVC, and risk rating for the transmission of vaccine-preventable diseases were compared, according to the two periods (2021 and 2022), using the McNemar test. Data regarding vaccination coverage were expressed in medians and interquartile ranges (IQR) since these data have a non-parametric distribution. The differences between the median vaccination coverage before and after the intervention were initially evaluated using the Mann-Whitney U Test, considering the IQR and a significance level of 5% for all immunobiologicals analyzed.

A significance level of 5% was adopted, and the statistical package Statistical Software for Professional (Stata), version 16.0, was used for data analysis.

Choropleth maps were also constructed to verify the spatial distribution of the risk rating of transmission of vaccine-preventable diseases for the 212 municipalities and eight priority RHA/RHS in the state of Minas Gerais. The QGIS program, version 2.18.14, was used for this analytical procedure.

### Ethical Aspects

The research was approved by the Ethics Committee of the Federal University of Minas Gerais under protocol CAAE 58407122.4.0000.5149.

## RESULTS

Concerning vaccination coverage, the highest percentage increase was observed in the chickenpox vaccine (16.81%; 82.92% in 2021 to 96.93% in 2022), followed by the triple viral D2 vaccine (14.57%; 70.99% in 2021 to 81.33% in 2022). The smallest increase in percentage terms was observed in vaccines against yellow fever (an increase of 1.18%; 84.55% in 2021 to 85.55% in 2022) and rotavirus (an increase of 5.71%; 86.03% in 2021 to 90.94% in 2022). Only the yellow fever vaccine demonstrated no statistical significance when comparing the two years (p = 0.264) (Table 1).

**Table 1:** Vaccination coverage in children under two years of age before and after the intervention of the research-action project in priority municipalities, Minas Gerais, 2021–2022.

Immunobiological	Year		p-value	Increase (%)
	2021	2022		
	Median (IQR)	Median (IQR)		
Rotavirus	86.03 (70.00–100.00)	90.94 (79.42–100.00)	< 0.001	5.71
Meningococcus C	86.07 (70.20–100.00)	93.12 (79.29–100.00)	< 0.001	8.19
Pneumococcal	84.84 (71.10–100.00)	94.42 (82.58–100.00)	< 0.001	11.29
Penta (DTP/Hib/HB)	84.72 (70.25–100.00)	91.45 (78.69–100.00)	< 0.001	7.94
Polio	84.70 (69.93–100.00)	92.34 (79.29–100.00)	< 0.001	9.02
Triple viral D1	88.74 (75.07–100.00)	94.92 (83.33–100.00)	< 0.001	6.96
Yellow fever	84.55 (65.50–98.26)	85.55 (72.33–98.97)	0.264	1.18
Triple viral D2	70.99 (47.07–89.76)	81.33 (66.17–100.00)	< 0.001	14.57
Hepatitis A	84.19 (68.18–100.00)	93.02 (81.59–100.00)	< 0.001	10.49
Chickenpox	82.98 (66.79–99.52)	96.93 (81.98–100.00)	< 0.001	16.81

IQR: interquartile ranges.

**Figure 1:**
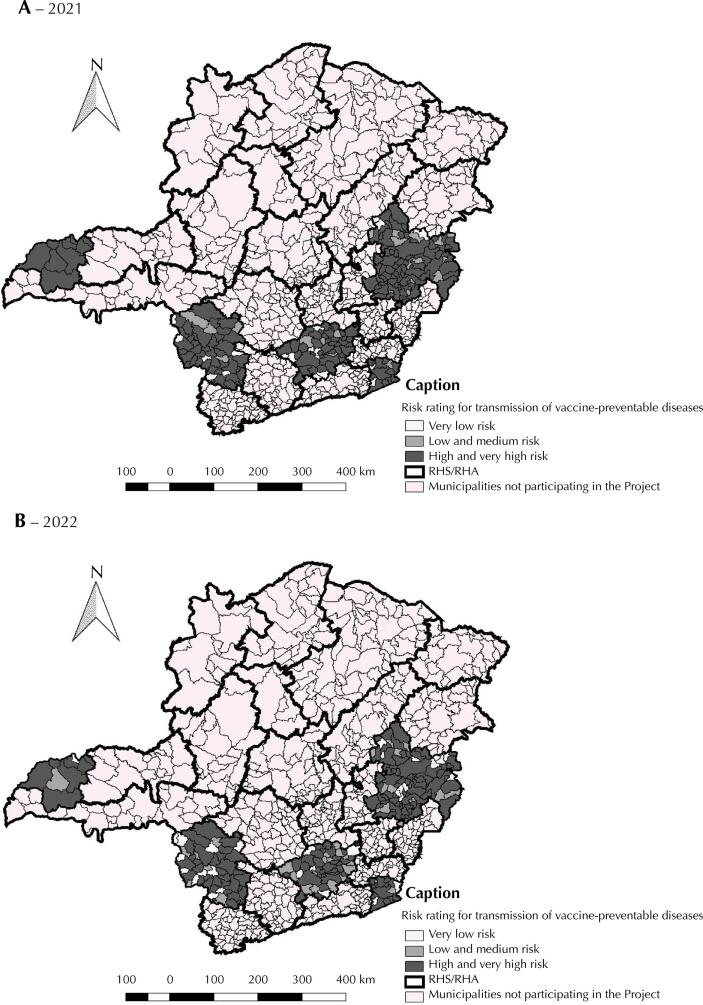
Spatial distribution of municipalities participating in the research-action project according to risk rating for transmission of vaccine-preventable diseases. Minas Gerais, Brazil. Note: (A) year 2021, before the intervention; (B) year 2022, after the intervention.

All immunization indicators increased after carrying out the action research project in the 212 municipalities and eight RHA/RHS participants. Regarding the “adequate” HVC rating (≥ 75% to ≤ 100%), there was an increase from 19.34% (2021)U to 37.60% (2022), with statistical significance (p = 0.022). Regarding “high” DR, only the oral human rotavirus vaccine showed a statistically significant reduction (p < 0.001) in this rating (16.04% in 2021 to 6.60% in 2022). According to the risk rating for the transmission of vaccine-preventable diseases, in 2021, 80.66% of the 212 municipalities were classified as “high and very high” risk. In 2022, after the intervention in the municipalities, there was a reduction in this rate to 68.40%, also statistically significant (p = 0.039) (Table 2, Figure 1A and 1B)

**Table 2:** Dropout rate, homogeneity of vaccination coverage, and risk rating for the transmission of vaccine-preventable diseases before and after the intervention of the research-action project in priority municipalities, Minas Gerais, 2021–2022.

Variable	Year		p-value
	2021	2022	
	n (%)	n (%)	
HVC (%)			0.022
Adequate (≥ 75% to ≤ 100%)	41 (19.34)	67 (31.60)	
Low (≥ 50% to < 75%)	36 (16.98)	34 (16.04)	
Very low (≥ 0% to < 50%)	135 (63.68)	111 (52.36)	
DR (%)			
Rotavirus oral vaccine			< 0.001
Low (< 5%)	142 (66.98)	175 (82.55)	
Medium (≥ 5% to ≤ 10%)	36 (16.98)	23 (10.85)	
High (> 10%)	34 (16.04)	14 (6.60)	
Pneumococcal disease vaccine			0.135
Low (< 5%)	144 (67.92)	158 (74.53)	
Medium (≥ 5% to ≤ 10%)	33 (15.57)	30 (14.15)	
High (> 10%)	35 (16.51)	24 (11.32)	
Pentavalent and hexavalent vaccine			0.502
Low (< 5%)	136 (64.15)	129 (60.85)	
Medium (≥ 5% to ≤ 10%)	29 (13.68)	39 (18.40)	
High (> 10%)	47 (22.17)	44 (20.75)	
Polio vaccine			0.921
Low (< 5%)	128 (60.38)	129 (60.85)	
Medium (≥ 5% to ≤10%)	32 (15.09)	37 (17.45)	
High (> 10%)	52 (24.53)	46 (21.70)	
Risk rating			0.039
Very low		31 (14.62)	
Low and medium		36 (16.98)	
High and very high		145 (68.40)	

n = number of municipalities; DR: dropout rate; HVC: homogeneity of vaccination coverage.

In 2021, 94.6% of children were in municipalities classified as “high or very high” risk for transmitting vaccine-preventable diseases. However, in 2022, there was a reduction (from 94.6% to 87.07%) in this proportion, showing the intervention’s modest effectiveness in question (Table 3).

**Table 3:** Proportion of children living in priority RHA/RHS according to risk rating for transmission of vaccine-preventable diseases in the state of Minas Gerais, Brazil, 2021 and 2022.

RHA/RHS	Total number of children	Very low		Low and medium		High and very high	
		n	%	n	%	n	%
2021							
Alfenas	4,971	477	9.6	160	3.2	4,334	87.2
Barbacena	6,042	110	1.8	137	2.3	5,795	95.9
Coronel Fabriciano	10,333	-	-	273	2.6	10,060	97.4
Governador Valadares	8,571	136	1.6	642	7.5	7,793	90.9
Ituiutaba	2,038	-	-	-	-	2,038	100
Leopoldina	2,525	61	2.4	38	1.5	2,426	96.1
Passos	5,445	107	2	234	4.3	5,104	93.7
São João Del Rei	2,703	287	10.6	89	3.3	2,327	86.1
Total 2021	42,628	1,178	2.76	1,573	3.69	39,877	93.55
2022							
Alfenas	4,996	479	9.59	281	5.62	4,236	84.79
Barbacena	5,805	175	3.01	919	15.83	4,711	81.15
Coronel Fabriciano	9,764	134	1.37	625	6.4	9,005	92.23
Governador Valadares	8,267	479	5.79	364	4.4	7,424	89.8
Ituiutaba	1,898	11	0.58	33	1.74	1,854	97.68
Leopoldina	2,378	75	3.15	151	6.35	2,152	90.5
Passos	5,366	700	13.05	273	5.09	4,393	81.87
São João Del Rei	2,787	227	8.14	409	14.68	2,151	77.18
Total 2022	41,261	2,280	7.4	3,055	7.4	35,926	87.07

RHA: Regional Health Administrations; RHS: Regional Health Superintendencies; SINASC: Live Birth Information System.

Note: population source: SINASC 2019 (for data for 2021) and SINASC 2020 (for data for 2022).

## DISCUSSION

The state research-action project was a before-after community clinical trial conducted in 212 municipalities belonging to eight RHA/RHS in Minas Gerais, Brazil.

According to Pinto et al.^17^, evaluating the impact of social strategies requires including analysis, monitoring, and management of their consequences, whether intended or not, positive or negative. These social interventions can be public policies, plans, projects, or businesses and include any change processes brought about by them.

Through a joint analysis of VC, HVC, and DR indicators in children under two years of age, this study showed that the state research-action project may have contributed to the reduction and change in the scenario of risk rating of transmission of vaccine-preventable diseases in Minas Gerais, Brazil.

Given the numerous challenges in controlling vaccine-preventable diseases, it is possible to reaffirm the need for actions that promote or rescue vaccination, to value the assertions proposed by the PNI, with a guarantee of maintaining high and homogeneous vaccination coverage throughout the national territory^1,4^.

The World Health Organization (WHO) guides the consolidation of immunization programs to legitimize innovative measures to strengthen collective immunity against vaccine-preventable diseases^6^. Successful immunization programs are essential strategies, resulting in the reduction, control, and eradication of vaccine-preventable diseases, directly leading to the reduction of child mortality^5,6^. However, since 2016, there has been a drop in vaccination coverage in Minas Gerais and other Brazilian states^1,4,7^. According to the data identified in this study, in 2021 and 2022, there was an increase in vaccination coverage in RHA/RHS in Minas Gerais after the implementation of the state project entitled “*Estratégias para o Aumento de Coberturas Vacinais nas Crianças Menores de 2 anos no Estado de Minas Gerais, Brasil: uma Pesquisa-Ação*” (Strategies for Increasing Vaccination Coverage in Children Under Two Years of Age in the State of Minas Gerais, Brazil: a Research-Action).

Among the ten vaccines included in the analyses, nine showed a statistically significant increase in vaccination coverage after workshops were held in the RHA/RHS host municipalities. Therefore, the notable importance of actions to eradicate, eliminate, or control vaccine-preventable diseases stands out, focusing on training and updating health professionals who work in vaccination rooms^1^ and reorganizing work process indicators, considering the municipal specificities.

One can highlight, for example, the importance of intensifying training, given the epidemiological relevance and records of vaccine failures for attenuated viral vaccines^18^. A systematic review in Hong Kong indicates that healthcare professionals’ guidance facilitates vaccine acceptance. Therefore, it is essential to have qualified professionals who know how to advise and direct vaccines and answer the community’s questions^19^.

Furthermore, regarding the results of this research, concerning the homogeneity rate (HVC), there was an increase in the “adequate” category (≥ 75% to < 100%) and a reduction in the “low” (≥ 50% to < 75% ) and “very low” (≥ 0% to < 50%) categories, after carrying out the state research-action procedures. Reducing morbidity and mortality from vaccine-preventable diseases will only be possible if coverage indicators are kept high and necessarily homogeneous^20^.

Regarding the DR of the human rotavirus oral vaccine, it was evident that, with the project workshops, there was a decrease in the “medium” and “high” rating and a statistically significant increase in the “low” classification. High DRs are a reality in Minas Gerais and some regions of Brazi^21^, a state with a sizeable territorial extension and very heterogeneous socioeconomic conditions in the municipalities. Several challenges to reducing DR in Brazilian municipalities^1,20^, and socioeconomic and environmental factors hinder the population’s equal access to vaccination rooms, contributing to high DR in the vaccination schedule^6,21^. Concerning the other vaccines that showed no change in DR, the justification may have been the concern of parents or guardians with the pain and suffering of children—inherent to administering vaccines with needles^24^—different from immunization against human rotavirus, which is administered orally.

Coordination between states and municipalities, together with the Ministry of Health, is a priority given the urgent need to maintain the quality and efficiency of the PNI, especially given the drop in vaccination coverage in children under two years of age^1^. Concerning the risk classification for the transmission of vaccine-preventable diseases according to RHA/RHS, there was an increase in the percentage of children living in territories classified as “very low,” “low,” and “medium” after carrying out the actions of this state project. The high and very high rating in Minas Gerais in the study by Silva et al.^25^, i.e., in the 853 municipalities, was at 80.9% in 2021, similar to data found in this study before the implementation of the research-action project.

Furthermore, the risk rating for the transmission of vaccine-preventable diseases is an important mechanism to assist managers in defining places that most urgently need interventions to optimize and improve vaccination coverage^13^.

Finally, this study presents some limitations inherent to studies with secondary databases. The available data is made available via SIPNI, and the researchers had no control over the quality of filling out the forms in this system. Furthermore, there is a possibility of underestimation of the data found due to errors in the system records. However, it is noteworthy that the system has solid foundations, capable of supporting health strategies and policies based on monitoring vaccination coverage in Brazil.

The study also presents some limitations inherent to the research-action approach. This project had a situational and specific objective, adapted to the reality and local circumstances of the RHA/RHS of Minas Gerais. Furthermore, the researchers did not exercise absolute control over all variables since these developed in a real context, with professionals equipped with autonomy and playing roles linked to care practices. Also noteworthy is the possibility of under-registration in the private network for the release of administered vaccine doses, which may affect the accuracy of the data analyzed.

As this study is uncontrolled, factors other than the intervention may have changed the outcomes. Another limitation is that the increase in vaccination coverage observed in 2022 may be related to the previous reduction in coverage due to the pandemic, followed by the search to update children’s vaccination records after the pandemic or trans-pandemic period.

Finally, as potentialities of this work, one can mention the engagement of interested parties—the active and collaborative involvement of healthcare managers, professionals in the field, and communities—which possibly contributed to the success of the research-action. Also noteworthy is the adequate infrastructure of most RHA/RHS, with well-equipped vaccination stations and trained professionals, which can facilitate the application of vaccines and improve vaccination coverage rates.

The socioeconomic and epidemiological variables of the regions in which the RHA/RHS are located can also be discussed, as it is believed that levels of education, income, and access to health services can affect adherence to vaccines. Furthermore, if there are outbreaks or cases of disease, the project’s actions may have a more significant impact on the prevention and control of these diseases in a RHA/RHS compared with other regions.

## CONCLUSION

Carrying out action research can value the active and collaborative participation of everyone involved in developing plans to bring together researchers, healthcare professionals, local communities, and all parties interested in expanding vaccination coverage. Such collaboration between different actors can, therefore, increase the acceptance and commitment of communities concerning strategies to encourage immunization, aiming to reduce the risk of occurrence and reintroduction of vaccine-preventable diseases in contexts stressed by social inequalities, such as the children’s audience.

It is essential to encourage projects such as the one presented in this article, which support the development of strategies applicable to the unique reality of each municipality and the precise location of specific groups without adequate vaccination protection.
