# Erratum

**DOI:** 10.11606/s1518-8787.2024058005484err

**Published:** 2024-04-25

In the article “Impact of a research-action on vaccination indicators in the state of Minas Gerais, Brazil”, DOI https://doi.org/10.11606/s1518-8787.2024058005484, published on the Revista de Saúde Pública. 2024;58:09, RSP corrects:

**Affiliation (page 1):**

Where it reads:

^II^Secretária de Estado da Saúde de Minas Gerais,

It should read:

^II^Secretaria de Estado da Saúde de Minas Gerais.

**Table 2 (page 6):**

**Table 2:** Dropout rate, homogeneity of vaccination coverage, and risk rating for the transmission of vaccine-preventable diseases before and after the intervention of the research-action project in priority municipalities, Minas Gerais, 2021–2022.

Variable	Year		p-value

	2021	2022	

	n (%)	n (%)	
HVC (%)			0.022
Adequate (≥ 75% to ≤ 100%)	41 (19.34)	67 (31.60)	
Low (≥ 50% to < 75%)	36 (16.98)	34 (16.04)	
Very low (≥ 0% to < 50%)	135 (63.68)	111 (52.36)	
DR (%)			
Rotavirus oral vaccine			< 0.001
Low (< 5%)	142 (66.98)	175 (82.55)	
Medium (≥ 5% to ≤ 10%)	36 (16.98)	23 (10.85)	
High (> 10%)	34 (16.04)	14 (6.60)	
Pneumococcal disease vaccine			0.135
Low (< 5%)	144 (67.92)	158 (74.53)	
Medium (≥ 5% to ≤ 10%)	33 (15.57)	30 (14.15)	
High (> 10%)	35 (16.51)	24 (11.32)	
Pentavalent and hexavalent vaccine			0.502
Low (< 5%)	136 (64.15)	129 (60.85)	
Medium (≥ 5% to ≤ 10%)	29 (13.68)	39 (18.40)	
High (> 10%)	47 (22.17)	44 (20.75)	
Polio vaccine			0.921
Low (< 5%)	128 (60.38)	129 (60.85)	
Medium (≥ 5% to ≤10%)	32 (15.09)	37 (17.45)	
High (> 10%)	52 (24.53)	46 (21.70)	
Risk rating			0.039
Very low	19 (8.96)	31 (14.62)	
Low and medium	22 (10.38)	36 (16.98)	
High and very high	171 (80.66)	145 (68.40)	

n = number of municipalities; DR: dropout rate; HVC: homogeneity of vaccination coverage.

In the column Year 2021, in the lines Very low, Low and medium, and High and very high, where it reads:

It should read:

**Table 2:** Dropout rate, homogeneity of vaccination coverage, and risk rating for the transmission of vaccine-preventable diseases before and after the intervention of the research-action project in priority municipalities, Minas Gerais, 2021–2022.

Variable	Year		p-value

	2021	2022	

	n (%)	n (%)	
HVC (%)			0.022
Adequate (≥ 75% to ≤ 100%)	41 (19.34)	67 (31.60)	
Low (≥ 50% to < 75%)	36 (16.98)	34 (16.04)	
Very low (≥ 0% to < 50%)	135 (63.68)	111 (52.36)	
DR (%)			
Rotavirus oral vaccine			< 0.001
Low (< 5%)	142 (66.98)	175 (82.55)	
Medium (≥ 5% to ≤ 10%)	36 (16.98)	23 (10.85)	
High (> 10%)	34 (16.04)	14 (6.60)	
Pneumococcal disease vaccine			0.135
Low (< 5%)	144 (67.92)	158 (74.53)	
Medium (≥ 5% to ≤ 10%)	33 (15.57)	30 (14.15)	
High (> 10%)	35 (16.51)	24 (11.32)	
Pentavalent and hexavalent vaccine			0.502
Low (< 5%)	136 (64.15)	129 (60.85)	
Medium (≥ 5% to ≤ 10%)	29 (13.68)	39 (18.40)	
High (> 10%)	47 (22.17)	44 (20.75)	
Polio vaccine			0.921
Low (< 5%)	128 (60.38)	129 (60.85)	
Medium (≥ 5% to ≤10%)	32 (15.09)	37 (17.45)	
High (> 10%)	52 (24.53)	46 (21.70)	
Risk rating			0.039
Very low		31 (14.62)	
Low and medium		36 (16.98)	
High and very high		145 (68.40)	

n = number of municipalities; DR: dropout rate; HVC: homogeneity of vaccination coverage.

