# Supplementary material for: Impact of a research-action on vaccination indicators in the state of Minas Gerais, Brazil
Source: Rev Saude Publica. 2024 Mar 4;58:09. doi: 10.11606/s1518-8787.2024058005484 (PMC10926982; doi:10.11606/s1518-8787.2024058005484)
Supplement: Translation

# Translation: Impacto de uma pesquisa-ação nos indicadores vacinais em Minas Gerais

**Authors:** Janaina Fonseca Almeida Souza, Thales Philipe Rodrigues da Silva, Thais Moreira Oliveira, Aline Mendes Vimieiro, Antônia Maria da Silva Teixeira, Adriana Coelho Soares, Elice Eliane Nobre Ribeiro, Giselle Lima de Freitas, Eduarda Dantas Gaspar, Fernanda Penido Matozinhos

**Affiliations:** I Brasil Universidade Federal de Minas Gerais. Escolas de Enfermagem. Programa de Pós-graduação em Saúde e Enfermagem. Belo Horizonte, MG, Brasil; II Brasil Secretaria de Estado da Saúde de Minas Gerais, Belo Horizonte, MG, Brasil; III Brasil Universidade Federal de São Paulo. Escola Paulista de Enfermagem. Departamento de Enfermagem na Saúde da Mulher. São Paulo, SP, Brasil; IV Brasil Santa Casa de Belo Horizonte. Belo Horizonte, MG, Brasil; V Brasil Sociedade Brasileira de Imunizações. São Paulo, SP, Brasil; VI Brasil Universidade Federal de Minas Gerais. Escola de Enfermagem. Departamento de Enfermagem Materno-Infantil e Saúde Pública. Belo Horizonte, MG, Brasil

**Keywords:** Cobertura Vacinal, Gestão de Riscos, Criança, Avaliação do Impacto na Saúde

## Abstract

**OBJETIVO:**

Analisar o impacto do projeto estadual de pesquisa-ação nos indicadores de imunização (coberturas vacinais – CV, homogeneidade de cobertura vacinal – HCV, taxa de abandono – TA e classificação de risco) antes e após a intervenção em municípios e Gerências Regionais de Saúde/Superintendências Regionais de Saúde (GRS/SRS) prioritários.

**MÉTODOS:**

O projeto estadual de pesquisa-ação foi um ensaio clínico comunitário, do tipo antes-depois, realizado em 212 municípios pertencentes a oito GRS/SRS do estado de Minas Gerais, Brasil. Compuseram a amostra do estudo as SRS/GRS com tendência decrescente para a cobertura vacinal de rotina em crianças menores de 1 ano, no período de 2015 a 2020. Neste estudo, foram utilizados dados secundários de CV e TA de 10 imunobiológicos recomendados para crianças menores de 2 anos, no período de janeiro a dezembro de 2021 (período pré-intervenção, anterior ao projeto estadual de pesquisa-ação) e de janeiro a dezembro de 2022 (período pós-intervenção). As variáveis categóricas foram apresentadas em proporções e, inicialmente, realizou-se a comparação entre as de TA, HCV e a classificação de risco para a transmissão de doenças imunopreveníveis, segundo os dois períodos (2021 e 2022), utilizando-se o teste McNemar.

**RESULTADOS:**

Observou-se um aumento de todos os indicadores de imunização após a realização do projeto de pesquisa-ação. No ano de 2021, 80,66% dos municípios do estado tiveram a classificação de risco para transmissão de doenças imunopreveníveis como “alto e muito alto risco”. Em 2022, o valor foi reduzido para 68,40%.

**CONCLUSÕES:**

A classificação de risco para transmissão de doenças imunopreveníveis é um mecanismo importante para auxiliar os gestores na definição de prioridades. O projeto estadual de pesquisa-ação empregou um método que possibilitou a construção e a execução de planos de ação singulares a cada município, direcionando a melhoria dos indicadores de imunização no estado.

## INTRODUÇÃO

O Programa Nacional de Imunização (PNI) brasileiro, criado no ano de 1973, é coordenado pelo Ministério da Saúde, com coparticipação das secretarias estaduais e municipais de saúde^1,2^. É um dos programas de imunização mais reconhecidos e completos do mundo^1,2^, especialmente em virtude das estratégias coletivas e individuais utilizadas, que garantem o alcance de elevadas coberturas vacinais ao longo dos anos para a maioria dos imunobiológicos ofertados de maneira gratuita para a população^3,4^.

Ao longo dos anos, o PNI impactou significativamente na redução da morbimortalidade de doenças imunopreveníveis na população brasileira, por constituir-se numa política de saúde pública eficiente^2^. Nesse sentido, as ações de vacinação são consideradas como das mais bem-sucedidas intervenções custo-efetivas em saúde^5,6^, em todos os ciclos de vida (crianças, adultos, gestantes e idosos) e populações específicas (como povos indígenas)^1,3^.

Entretanto, observa-se no território brasileiro uma redução acentuada nas taxas de cobertura vacinal a partir do ano de 2016^7^, potencializada pela pandemia de covid-19^11,12^. Este cenário evidencia um grave problema para a imunidade coletiva e coloca a população em risco de transmissão de doenças imunopreveníveis^13^.

Em Minas Gerais – o quarto estado com a maior área territorial e o segundo em quantidade de habitantes, localizado na Região Sudeste do país^14^–, a queda da cobertura vacinal e aumento da quantidade de municípios em alto risco para transmissão de doenças imunopreveníveis seguiu tendência similar à dos demais estados do Brasil^10^, no período de 2015 a 2020. Considerando como unidade de análise as 28 Gerências Regionais de Saúde/Superintendências Regionais de Saúde (GRS/SRS) do estado de Minas Gerais, identificou-se a menor proporção de GRS/SRS que alcançaram as metas preconizadas de cobertura vacinal para os imunobiológicos estudados, no ano de 2020^11^.

A queda da cobertura vacinal é multifatorial^1^. Para superar os desafios das baixas coberturas, para além do diagnóstico situacional, é necessária a integração das diversas áreas do setor saúde, dos setores sociais e da educação, bem como a identificação de bolsões de indivíduos susceptíveis e o desenvolvimento de estratégias para garantir a manutenção de elevadas coberturas vacinais^1,9^.

Nessa perspectiva, e considerando a situação de vulnerabilidade das crianças, em 2021, o Núcleo de Estudos e Pesquisa em Vacinação, da Escola de Enfermagem da Universidade Federal de Minas Gerais (NUPESV-EEUFMG), articulou-se à Secretaria Estadual de Saúde do Estado de Minas Gerais (SES-MG) em um projeto pesquisa-ação intitulado: “Estratégias para o Aumento de Coberturas Vacinais nas Crianças Menores de Dois Anos no Estado de Minas Gerais, Brasil: uma Pesquisa-Ação”, visando melhorar a cobertura vacinal de crianças por meio da realização de oficinas e construção de planos de ação singulares à realidade de cada município do estado.

A pesquisa-ação (PA) refere-se a uma metodologia aplicada em várias disciplinas científicas, que abrange também a área da saúde, devido a sua capacidade de compreender profundamente um objeto de estudo e, ao mesmo tempo, aprimorar a prática associada a ele^15^. Com o intuito de compreender as fragilidades e potencialidades de cada município em relação à imunização de crianças com menos de 2 anos, bem como de promover um espaço para a troca de ideias e a construção coletiva de conhecimento, optou-se pela técnica da realização de oficinas. Sabe-se que, nessa metodologia, os participantes da pesquisa têm oportunidade de influenciar e modificar a realidade^15^. As oficinas oferecem a chance de negociação, debate argumentativo e diálogo entre os participantes, tornando-se, assim, uma ferramenta poderosa para envolver e transformar a realidade, uma vez que facilitam a integração de questões teóricas e metodológicas, bem como das implicações éticas e políticas^16^.

O objetivo deste estudo foi analisar o impacto do projeto estadual de pesquisa-ação nas coberturas vacinais (CV), especialmente no indicador de homogeneidade de cobertura vacinal (HCV), na taxa de abandono (TA) e na classificação de risco para transmissão de doenças imunopreveníveis nos municípios de Minas Gerais, Brasil, comparando os anos de 2022 e 2021.

## MÉTODOS

Compuseram a amostra do estudo as SRS/GRS com tendência decrescente para a cobertura vacinal de rotina em crianças menores de 1 ano no período de 2015 a 2020^10^, a saber: SRS Alfenas: 24 municípios; SRS Barbacena: 31 municípios; SRS Coronel Fabriciano: 35 municípios; SRS Governador Valadares: 51 municípios; GRS Ituiutaba: nove municípios; GRS Leopoldina: 15 municípios; SRS Passos: 27 municípios; e GRS São João Del Rey: 20 municípios; totalizando 212 municípios, ou seja, 24,85% do total do estado. As SRS/GRS que compuseram a amostra deste estudo foram selecionadas baseando-se no estudo prévio realizado por Souza et al.^10^ (2022), que identificou tendência decrescente para os imunobiológicos preconizados para menores de 2 anos em pelo menos cinco destas gerências regionais.

As oficinas tiveram duração de 12 horas, foram realizadas em diferentes contextos e conduzidas por profissionais da SES-MG e NUPESV-EEUFMG, exigindo uma organização prévia detalhada. A cooperação entre ambas as partes foi fundamental desde a criação do projeto de pesquisa até a colaboração contínua. Para operacionalização do projeto, foram realizadas oficinas com profissionais da atenção primária à saúde (especialmente enfermeiros), técnicos e auxiliares de enfermagem que trabalham diretamente com a vacinação nos municípios, gestores, coordenadores da vigilância epidemiológica e atenção primária à saúde e secretários de saúde, além de outros parceiros externos (representantes de universidades, Conselhos Municipais de Saúde, Conselho de Secretários Municipais de Saúde). As oficinas foram operacionalizadas com as seguintes etapas: 1. Exposição dialogada: análise de coberturas vacinais em crianças menores de 2 anos nos municípios e nas GRS/SRS, roda de conversa e *brainstorming* com perguntas disparadoras para problematização; 2. Início das atividades de grupo e construção dos Planos de Ação Municipais; 3. Apresentação, por meio de um relator de cada grupo, das discussões que foram realizadas para início da construção dos planos de ação.

Após a realização das oficinas, cada município participante tinha 15 dias para enviar seu plano de ação para a respectiva GRS/SRS, após pactuação com gestores, equipe técnica e equipe assistencial e aprovação pelo Conselho Municipal de Saúde. Para elaboração dos planos, foram considerados os seguintes eixos estratégicos: gestão de pessoas, comunicação social, parcerias estratégicas, infraestrutura e logística, articulação gestora e monitoramento e avaliação. As oficinas ocorreram entre março e junho de 2022, com 515 participantes, incluindo gestores de saúde, coordenadores de vigilância em saúde e parceiros externos. Cada município teve, em média, quatro representantes nas oficinas, que foram conduzidas por uma equipe coordenadora.

Neste estudo, foram utilizados dados secundários de CV e TA de dez imunobiológicos recomendados para crianças menores de 2 anos, em 2021 (período pré-intervenção, anterior ao projeto estadual de pesquisa-ação) e em 2022 (período pós-intervenção).

Os imunobiológicos recomendados para crianças menores de 2 anos, em ambos os anos (2021 e 2022), foram: vacina oral contra o rotavírus (2ª dose da vacina rotavírus no Sistema Único de Saúde (SUS) mais a 2ª dose da rota-pentavalente na rede privada), vacina contra doença meningocócica C (2ª dose meningocócica C e a 2ª dose meningocócica ACWY), vacina contra doença pneumocócica (2ª dose da pneumocócica 10V e 2ª dose da pneumocócica 13V), vacina pentavalente (3ª dose da vacina pentavalente mais a 3ª dose da vacina hexavalente na rede privada), vacina contra a poliomielite (3ª dose da VIP, VOP, pentavalente da rede privada e a hexavalente também na rede privada), vacina contra febre amarela (dose única, dose inicial e a 1ª dose), 1ª dose da vacina tríplice viral (1ª dose da tríplice viral, 1ª dose de quádrupla viral e 1º dose da tetra viral), 2ª dose da vacina tríplice viral (2ª dose da tríplice viral, 2ª dose da quádrupla viral, 2ª dose e dose única da tetra viral), vacina contra hepatite A (considerou-se a 1ª dose) e vacina contra varicela (1ª dose varicela e a 1 ª dose tetra viral).

Todas as informações foram extraídas do Sistema de Informações do Programa Nacional de Imunizações (SIPNI), disponível em: <sipni.datasus.gov.br>. Não foram avaliadas as vacinas BCG e Hepatite B, pois estas são administradas, em sua maioria, nas maternidades, o que poderia causar um viés nas análises.

Para as análises de 2021, a CV foi calculada utilizando como denominador a população do Sistema de Informações sobre Nascidos Vivos (SINASC) menores de um ano de 2019. Para 2022, foi utilizado o SINASC referente ao ano de 2020, sempre considerando a informação mais atualizada. No numerador, utilizou-se as doses (dose imunizante ou a que completa o esquema vacinal) aplicadas por faixa-etária, e imunobiológico, conforme Calendário Nacional de Vacinação do Ministério da Saúde.

As taxas das CV foram categorizadas segundo as metas estabelecidas pelo PNI (maior ou igual a 90% para a vacina oral de rotavírus humano e maior ou igual a 95% para os demais imunobiológicos) em: “muito baixa” (< 50%), “baixa” (≥ 50 % e menor que a meta) e “adequada” (≥ que a meta)^13^.

Verificou-se, também, a avaliação da homogeneidade de cobertura vacinal (HCV) entre as vacinas analisadas, seguindo-se a definição adotada por estudo prévio de Braz et al*.*^13^, pactuado pelo Sistema Único de Saúde (SUS), por meio do Contrato Organizativo de Ação Pública da Saúde (COAP): “adequada” quando apresentaram HCV de ≥ 75% a ≤ 100% para as dez vacinas com cobertura adequada (≥ que a meta), “baixa” quando apresentaram de ≥ 50% a < 75 e “muito baixa” quando a porcentagem foi < 50% para as dez vacinas analisadas.

Calculou-se, também, a TA para as vacinas multidose, sendo: vacina contra a doença meningocócica C (para o ano de 2022), vacina pentavalente (para os anos de 2021 e 2022), vacina contra a doença pneumocócica (para os anos de 2021 e 2022), vacina contra a poliomielite (para os anos de 2021 e 2022) e vacina oral de rotavírus humano (para os anos de 2021 e 2022), sendo classificada em: “baixa” TA (< 5%), “média” TA (≥ 5% a < 10%) e “alta” TA (≥ 10%)^13^. Para o cálculo da TA, considerou-se a diferença entre o número de primeiras e de últimas doses do esquema vacinal aplicado, dividido pelo número das primeiras doses aplicadas, multiplicando-se o resultado por 100^13^.

Os municípios foram categorizados com base em seus tamanhos populacionais, conforme definido previamente no estudo de Braz et al.^13^ Essa classificação considera três grupos: municípios de pequeno porte, que têm uma população igual ou inferior a 20 mil habitantes; municípios de médio porte, com população variando entre 20.001 e 100 mil habitantes; e municípios de grande porte, com população igual ou superior a 100.001 habitantes.

Por fim, os municípios participantes da pesquisa foram classificados de acordo com o risco de transmissão de doenças imunopreveníveis em cinco estratos, segundo Braz et al.^13^, para os dois anos de análise:

•“Muito baixo”: municípios com HCV = 100%;
•“Baixo”: municípios com HCV de ≥ 75% a < 100%, com CV adequada para as vacinas poliomielite, tríplice viral (compromisso internacional de eliminação de doenças) e, ainda, a vacina pentavalente, considerada como “marcador padrão” de qualidade de serviço de vacinação (esquema de três doses injetáveis);
•“Médio”: município com HCV ≥ 75% e < 100% e CV abaixo da meta para uma ou mais das vacinas poliomielite, tríplice viral ou pentavalente;
•“Alto”: municípios com HCV < 75%, independente da cobertura vacinal;
•“Muito alto”: município com HCV < 75%, alta TA (≥ 10%) para qualquer uma das vacinas avaliadas e com grande porte populacional, bem como os municípios sem registro de vacinação para qualquer vacina, independentemente do porte populacional.

Devido ao número pequeno de municípios classificados como “médio” e “muito alto risco” para transmissão de doenças imunopreveníveis, essas categorias foram agrupadas em “baixo e médio risco” e “alto e muito alto risco” de transmissão de doenças imunopreveníveis.

As variáveis categóricas foram apresentadas em proporções e, inicialmente, realizou-se a comparação dos valores da TA, HCV e classificação de risco para a transmissão de doenças imunopreveníveis, segundo os dois períodos (2021 e 2022), utilizando-se o teste McNemar. Os dados referentes às coberturas vacinais foram expressos em medianas e intervalos interquartis (IIQ), uma vez que esses dados têm uma distribuição não paramétrica. Inicialmente, as diferenças entre a mediana da cobertura vacinal antes e após a intervenção foram avaliadas por meio do Teste de U de Mann-Whitney, considerando-se o IIQ e um nível de significância de 5% para todos os imunobiológicos analisados.

Adotou-se o nível de significância de 5% e, para a análise dos dados, foi utilizado o pacote estatístico Statistical Software for Professional (Stata), versão 16.0.

Também foram construídos mapas coropléticos para verificar a distribuição espacial da classificação do risco de transmissão de doenças imunopreveníveis para os 212 municípios e oito GRS/SRS prioritárias do estado de Minas Gerais. Para este procedimento analítico, utilizou-se o programa QGIS, versão 2.18.14.

### Aspectos Éticos

A pesquisa foi aprovada pelo Comitê de Ética da Universidade Federal de Minas Gerais, sob o protocolo CAAE 58407122.4.0000.5149.

## RESULTADOS

Em relação às coberturas vacinais, observou-se maior percentual de aumento na vacina varicela (16,81%; 82,92% em 2021 para 96,93% em 2022), seguida da vacina tríplice viral D2 (14,57%; 70,99% em 2021 para 81,33% em 2022). O menor aumento em termos percentuais foi observado nas vacinas contra a febre amarela (aumento de 1,18%; 84,55% em 2021 para 85,55% em 2022) e rotavírus (aumento de 5,71%; 86,03% em 2021 para 90,94% em 2022). Apenas a vacina contra a febre amarela não demonstrou significância estatística ao comparar os dois anos (p = 0,264) (Tabela 1).

**Tabela 1:** Cobertura vacinal em crianças menores de 2 anos de idade antes e após a intervenção do projeto pesquisa-ação em municípios prioritários, Minas Gerais, 2021–2022.

Imunobiológico	Ano		p-valor	Aumento(%)
	2021	2022		
	Mediana (IIQ)	Mediana (IIQ)		
Rotavírus	86,03 (70,00–100,00)	90,94 (79,42–100,00)	< 0,001	5,71
Meningococo C	86,07 (70,20–100,00)	93,12 (79,29–100,00)	< 0,001	8,19
Pneumocócica	84,84 (71,10–100,00)	94,42 (82,58–100,00)	< 0,001	11,29
Penta (DTP/Hib/HB)	84,72 (70,25–100,00)	91,45 (78,69–100,00)	< 0,001	7,94
Poliomielite	84,70 (69,93–100,00)	92,34 (79,29–100,00)	< 0,001	9,02
Tríplice viral D1	88,74 (75,07–100,00)	94,92 (83,33–100,00)	< 0,001	6,96
Febre amarela	84,55 (65,50–98,26)	85,55 (72,33–98,97)	0,264	1,18
Tríplice viral D2	70,99 (47,07–89,76)	81,33 (66,17–100,00)	< 0,001	14,57
Hepatite A	84,19 (68,18–100,00)	93,02 (81,59–100,00)	< 0,001	10,49
Varicela	82,98 (66,79–99,52)	96,93 (81,98–100,00)	< 0,001	16,81

IIQ: intervalos interquartis.

Observou-se um aumento de todos os indicadores de imunização após a realização do projeto de pesquisa-ação nos 212 municípios e oito GRS/SRS participantes. Em relação à classificação de “adequada” HCV (≥ 75% a ≤ 100%), houve um aumento de 19,34% (2021), para 37,60% (2022), com significância estatística (p = 0,022). No que tange à “alta” TA, somente a vacina oral de rotavírus humano apresentou redução estatisticamente significativa (p < 0,001) nessa classificação (16,04% em 2021, para 6,60% em 2022). Segundo a classificação de risco para a transmissão de doenças imunopreveníveis, no ano de 2021, 80,66% dos 212 municípios foram classificados como “alto e muito alto risco”. Em 2022, após a intervenção nos municípios, observou-se redução dessa taxa para 68,40%, também estatisticamente significativa (p = 0,039) (Tabela 2, Figura A e Figura B)

**Tabela 2:** Taxa de abandono, homogeneidade de cobertura vacinal e classificação de risco para a transmissão de doenças imunopreveníveis antes e após a intervenção do projeto pesquisa-ação em municípios prioritários, Minas Gerais, 2021–2022.

Variável	Ano		p-valor
	2021	2022	
	n (%)	n (%)	
HCV (%)			0,022
Adequada (≥ 75% a ≤ 100%)	41 (19,34)	67 (31,60)	
Baixa (≥ 50% a < 75%)	36 (16,98)	34 (16,04)	
Muito baixa (≥ 0% a < 50%)	135 (63,68)	111 (52,36)	
TA (%)			
Vacina oral contra rotavírus			< 0,001
Baixa (< 5%)	142 (66,98)	175 (82,55)	
Média (≥ 5% a ≤ 10%)	36 (16,98)	23 (10,85)	
Alta (> 10%)	34 (16,04)	14 (6,60)	
Vacina contra doença pneumocócica			0,135
Baixa (< 5%)	144 (67,92)	158 (74,53)	
Média (≥ 5% a ≤ 10%)	33 (15,57)	30 (14,15)	
Alta (> 10%)	35 (16,51)	24 (11,32)	
Vacina pentavalente e hexavalente			0,502
Baixa (< 5%)	136 (64,15)	129 (60,85)	
Média (≥ 5% a ≤ 10%)	29 (13,68)	39 (18,40)	
Alta (> 10%)	47 (22,17)	44 (20,75)	
Vacina contra a poliomielite			0,921
Baixa (< 5%)	128 (60,38)	129 (60,85)	
Média (≥ 5% a ≤10%)	32 (15,09)	37 (17,45)	
Alta (> 10%)	52 (24,53)	46 (21,70)	
Classificação de risco			0,039
Muito baixo		31 (14,62)	
Baixo e médio		36 (16,98)	
Alto e muito alto		145 (68,40)	

n = número de municípios; TA: taxa de abandono; HCV: homogeneidade de cobertura vacinal.

**Figura:** Distribuição espacial dos municípios participantes do projeto pesquisa-ação segundo classificação de risco para transmissão de doenças imunopreveníveis. Minas Gerais, Brasil. Nota: (A) ano de 2021, antes da intervenção; (B) ano de 2022, depois da intervenção.

No ano de 2021, 94,6% das crianças encontravam-se em municípios classificados como de “alto ou muito alto risco” para transmissão de doenças imunopreveníveis. Porém, em 2022, observou-se redução (de 94,6%, em 2021, para 87,07%, em 2022) nessa proporção, evidenciando uma efetividade modesta da intervenção em questão (Tabela 3).

**Tabela 3:** Proporção de crianças residentes em GRS/SRS prioritárias segundo classificação de risco para transmissão de doenças imunopreveníveis no estado de Minas Gerais, Brasil, 2021 e 2022.

GRS/SRS	Número total de crianças	Muito baixo		Baixo e médio		Alto e muito alto	
		n	%	n	%	n	%
2021							
Alfenas	4.971	477	9,6	160	3,2	4.334	87,2
Barbacena	6.042	110	1,8	137	2,3	5.795	95,9
Coronel Fabriciano	10.333	-	-	273	2,6	10.060	97,4
Governador Valadares	8.571	136	1,6	642	7,5	7.793	90,9
Ituiutaba	2.038	-	-	-	-	2.038	100
Leopoldina	2.525	61	2,4	38	1,5	2.426	96,1
Passos	5.445	107	2	234	4,3	5.104	93,7
São João Del Rei	2.703	287	10,6	89	3,3	2.327	86,1
Total 2021	42.628	1.178	2,76	1.573	3,69	39.877	93,55
2022							
Alfenas	4.996	479	9,59	281	5,62	4.236	84,79
Barbacena	5.805	175	3,01	919	15,83	4.711	81,15
Coronel Fabriciano	9.764	134	1,37	625	6,4	9.005	92,23
Governador Valadares	8.267	479	5,79	364	4,4	7.424	89,8
Ituiutaba	1.898	11	0,58	33	1,74	1.854	97,68
Leopoldina	2.378	75	3,15	151	6,35	2.152	90,5
Passos	5.366	700	13,05	273	5,09	4.393	81,87
São João Del Rei	2.787	227	8,14	409	14,68	2.151	77,18
Total 2022	41.261	2.280	7,4	3.055	7,4	35.926	87,07

GRS: Gerências Regionais de Saúde; SRS: Superintendências Regionais de Saúde; SINASC: Sistema de Informações sobre Nascidos Vivos.

Nota: fonte da população: SINASC 2019 (para dados do ano de 2021) e SINASC 2020 (para dados do ano de 2022).

## DISCUSSÃO

O projeto estadual de pesquisa-ação foi um ensaio clínico comunitário, do tipo antes-depois, realizado em 212 municípios pertencentes a oito GRS/SRS de Minas Gerais, Brasil.

Segundo Pinto et al.^17^, ao avaliar o impacto de estratégias sociais é necessário incluir os processos de análise, monitoramento e gestão de suas consequências, pretendidas ou não, positivas ou negativas. Estas intervenções sociais podem ser políticas públicas, planos, projetos ou negócios e incluem quaisquer processos de mudança provocados por elas.

Este estudo evidenciou, por meio de uma análise conjunta dos indicadores de CV, HCV e TA em crianças menores de 2 anos, que o projeto estadual de pesquisa-ação pode ter contribuído para a redução e mudança do cenário da classificação de risco de transmissão de doenças imunopreveníveis em Minas Gerais, Brasil.

Diante dos inúmeros desafios para o controle das doenças imunopreveníveis, é possível reafirmar a necessidade de ações que promovam ou resgatem a vacinação, de forma a valorizar as assertivas propostas pelo PNI, com garantia da manutenção de altas e homogêneas coberturas vacinais em todo o território nacional^1,4^.

A Organização Mundial da Saúde (OMS) orienta a consolidação dos programas de imunização, de forma a legitimar medidas inovadoras para fortalecer a imunidade coletiva contra doenças imunopreveníveis^6^. Programas de imunização bem-sucedidos configuram-se como estratégias importantes, resultando na redução, controle e erradicação de doenças imunopreveníveis, o que culmina diretamente na redução da mortalidade infantil^5,6^. Entretanto, em Minas Gerais – bem como em outros estados brasileiros –, desde 2016, é experenciada uma queda das coberturas vacinais^1,4,7^. De acordo com os dados identificados neste estudo, observou-se que, nos anos de 2021 e 2022 houve aumento da cobertura vacinal nas GRS/SRS em Minas Gerais, após a realização do projeto estadual intitulado “Estratégias para o Aumento de Coberturas Vacinais nas Crianças Menores de 2 anos no Estado de Minas Gerais, Brasil: uma Pesquisa-Ação”.

Entre as dez vacinas incluídas nas análises, nove apresentaram aumento estatisticamente significativo da cobertura vacinal após a realização de oficinas nos municípios sede das GRS/SRS. Destaca-se, portanto, a notória importância das ações para erradicar, eliminar ou controlar doenças imunopreveníveis, com foco na capacitação e atualização dos profissionais de saúde que atuam nas salas de vacinação^1^, além da reorganização dos indicadores de processo de trabalho, considerando as especificidades municipais.

Pode-se ressaltar, por exemplo, a importância da intensificação de capacitações, dada a relevância epidemiológica e registros de falhas vacinais para vacinas virais atenuadas^18^. Revisão sistemática realizada em Hong Kong indica que a orientação do profissional de saúde é um facilitador de aceitação da vacina, dessa forma, é importante ter profissionais qualificados que saibam aconselhar e direcionar as vacinas, bem como sanar os questionamentos da comunidade^19^.

Ainda em relação aos resultados desta pesquisa, no que tange à taxa de homogeneidade (HCV), houve aumento da categoria “adequada” (≥ 75% a < 100%) e redução das categorias “baixa” (≥ 50% a < 75%) e “muito baixa” (≥ 0% a < 50%), após a realização das ações da pesquisa-ação estadual. A redução da morbidade e da mortalidade por doenças imunopreveníveis somente será possível se os indicadores de cobertura forem mantidos elevados e necessariamente homogêneos^20^.

Quanto à TA da vacina oral de rotavírus humano, evidenciou-se que, com a realização das oficinas do projeto, houve diminuição da classificação “média” e “alta” e incremento estatisticamente significativo da classificação “baixa”. As altas TA são realidade em Minas Gerais – bem como em algumas regiões do Brasil^21^–, estado com uma extensão territorial alta e condições socioeconômicas dos municípios bastante heterogêneas. Diversos são os desafios para reduzir a TA nos municípios brasileiros^1,20^, e fatores socioeconômicos e ambientais prejudicam o acesso de forma igualitária da população às salas de vacinas, o que pode contribuir para altas TA do esquema vacinal^6,21^. Em relação às demais vacinas que não apresentaram mudança na TA, a justificativa pode ter sido a preocupação dos pais ou responsáveis com a dor e o sofrimento das crianças inerente ao processo de administração de vacinas com agulhas^24^–, diferente da imunização contra o rotavírus humano, que é administrada por via oral.

A articulação entre os estados e municípios, em conjunto com o Ministério da Saúde, é uma prioridade diante da urgente necessidade de manutenção da qualidade e eficiência do PNI, especialmente perante a queda das coberturas vacinais em crianças menores de 2 anos^1^. Em relação à classificação de risco para a transmissão de doenças imunopreveníveis segundo GRS/SRS, houve aumento do percentual de crianças vivendo nos territórios classificados em “muito baixo”, “baixo” e “médio” após a realização das ações deste projeto estadual. A classificação de risco alto e muito alto em Minas Gerais no estudo de Silva et al.^25^, ou seja, nos 853 municípios, encontrava-se em 80,9% no ano de 2021, dado semelhante encontrado neste estudo, antes da implementação do projeto pesquisa-ação.

Ademais, a classificação de risco para transmissão de doenças imunopreveníveis é um mecanismo importante para auxiliar os gestores na definição de locais que necessitam com mais urgência de intervenções para otimização e melhoria das coberturas vacinais^13^.

Por fim, este estudo apresenta algumas limitações inerentes aos estudos com bases de dados secundárias. Os dados disponíveis são disponibilizados via SIPNI, e os pesquisadores não tiveram controle sobre a qualidade do preenchimento dos formulários deste sistema. Ademais, há possibilidade de subestimação dos dados encontrados, devido a falhas dos registros no sistema. Contudo, destaca-se que o sistema possui bases sólidas, sendo capaz de subsidiar estratégias e políticas de saúde a partir do monitoramento das coberturas vacinais no Brasil.

O estudo apresenta, também, algumas limitações inerentes à abordagem de pesquisa-ação. Este projeto teve um objetivo situacional e específico, adaptado à realidade e às circunstâncias locais das GRS/SRS de Minas Gerais. Ademais, os pesquisadores não exerceram controle absoluto sobre todas as variáveis, uma vez que estas se desenvolveram em um contexto real, com profissionais munidos de autonomia e desempenhando papéis atrelados às práticas assistenciais. Destaca-se, também, a possibilidade de sub-registro na rede privada de lançamento das doses de vacinas aplicadas, o que pode afetar a precisão dos dados analisados.

Trata-se de um estudo não controlado e, portanto, outros fatores além da intervenção podem ter mudado os desfechos. Outra limitação é a possibilidade de que o aumento na cobertura vacinal observado em 2022 possa estar relacionado à redução prévia da cobertura devido à pandemia, seguida pela busca de atualização das carteiras de vacinação das crianças após o período pandêmico ou transpandêmico.

Pode-se citar, por fim, como potencialidades deste trabalho, o engajamento das partes interessadas – o envolvimento ativo e colaborativo de gestores de saúde, profissionais da área e das comunidades –, que possivelmente contribuiu para o sucesso da pesquisa-ação. Destaca-se, também, a infraestrutura adequada da maioria das GRS/SRS, com postos de vacinação bem equipados e profissionais capacitados, o que pode facilitar a aplicação das vacinas e melhorar os índices de cobertura vacinal.

As variáveis de cunho socioeconômico e epidemiológicos no qual as GRS/SRS se localizam também pode ser discutida, pois acredita-se que níveis de educação, renda e acesso aos serviços de saúde podem afetar a adesão às vacinas. Além disso, caso haja surtos ou casos de doenças, as ações do projeto podem ter um impacto mais significativo na prevenção e controle dessas enfermidades em uma GRS/SRS quando comparada a outras regiões.

## CONCLUSÃO

A realização da pesquisa-ação pode valorizar a participação ativa e colaborativa de todos os envolvidos no processo de desenvolvimento de planos para aproximar pesquisadores, profissionais de saúde, comunidades locais e todas as partes interessadas em contribuir para a ampliação da cobertura vacinal. Tal colaboração entre diferentes atores pode, portanto, aumentar a aceitação e o comprometimento das comunidades em relação às estratégias de incentivo à imunização, visando a redução do risco de ocorrência e de reintrodução de doenças imunopreveníveis, em contextos tensionados pelas desigualdades sociais, como o do público infantil.

É imprescindível estimular projetos como o apresentado neste artigo, que subsidiem a elaboração de estratégias aplicáveis à realidade singular de cada município e a localização precisa de grupos específicos, que se encontram sem a proteção vacinal adequada.
