# Supplementary material for: Erratum
Source: Rev Saude Publica. 2024 Apr 25;58:09err. doi: 10.11606/s1518-8787.2024058005484err (PMC11090613; doi:10.11606/s1518-8787.2024058005484err)
Supplement: Translation

# Translation: Errata

No artigo “Impacto de uma pesquisa-ação nos indicadores vacinais em Minas Gerais”, DOI https://doi.org/10.11606/s1518-8787.2024058005484, publicado na Revista de Saúde Pública.2024;58:09, a RSP corrige:

**Afiliação (página 1):**

Onde se lê:

^II^Secretária de Estado da Saúde de Minas Gerais,

Leia-se:

^II^Secretaria de Estado da Saúde de Minas Gerais.

**Tabela 2 (página 6):**

**Tabela 2:** Taxa de abandono, homogeneidade de cobertura vacinal e classificação de risco para a transmissão de doenças imunopreveníveis antes e após a intervenção do projeto pesquisa-ação em municípios prioritários, Minas Gerais, 2021–2022.

Variável	Ano		p-valor

	2021	2022	

	n (%)	n (%)	
HCV (%)			0,022
Adequada (≥ 75% a ≤ 100%)	41 (19,34)	67 (31,60)	
Baixa (≥ 50% a < 75%)	36 (16,98)	34 (16,04)	
Muito baixa (≥ 0% a < 50%)	135 (63,68)	111 (52,36)	
TA (%)			
Vacina oral contra rotavírus			< 0,001
Baixa (< 5%)	142 (66,98)	175 (82,55)	
Média (≥ 5% a ≤ 10%)	36 (16,98)	23 (10,85)	
Alta (> 10%)	34 (16,04)	14 (6,60)	
Vacina contra doença pneumocócica			0,135
Baixa (< 5%)	144 (67,92)	158 (74,53)	
Média (≥ 5% a ≤ 10%)	33 (15,57)	30 (14,15)	
Alta (> 10%)	35 (16,51)	24 (11,32)	
Vacina pentavalente e hexavalente			0,502
Baixa (< 5%)	136 (64,15)	129 (60,85)	
Média (≥ 5% a ≤ 10%)	29 (13,68)	39 (18,40)	
Alta (> 10%)	47 (22,17)	44 (20,75)	
Vacina contra a poliomielite			0,921
Baixa (< 5%)	128 (60,38)	129 (60,85)	
Média (≥ 5% a ≤10%)	32 (15,09)	37 (17,45)	
Alta (> 10%)	52 (24,53)	46 (21,70)	
Classificação de risco			0,039
Muito baixo	19 (8,96)	31 (14,62)	
Baixo e médio	22 (10,38)	36 (16,98)	
Alto e muito alto	171 (80,66)	145 (68,40)	

n = número de municípios; TA: taxa de abandono; HCV: homogeneidade de cobertura vacinal.

Na coluna Ano 2021, nas linhas Muito baixo, Baixo e médio, Alto e muito alto, onde se lê:

Leia-se:

**Tabela 2:** Taxa de abandono, homogeneidade de cobertura vacinal e classificação de risco para a transmissão de doenças imunopreveníveis antes e após a intervenção do projeto pesquisa-ação em municípios prioritários, Minas Gerais, 2021–2022.

Variável	Ano		p-valor

	2021	2022	

	n (%)	n (%)	
HCV (%)			0,022
Adequada (≥ 75% a ≤ 100%)	41 (19,34)	67 (31,60)	
Baixa (≥ 50% a < 75%)	36 (16,98)	34 (16,04)	
Muito baixa (≥ 0% a < 50%)	135 (63,68)	111 (52,36)	
TA (%)			
Vacina oral contra rotavírus			< 0,001
Baixa (< 5%)	142 (66,98)	175 (82,55)	
Média (≥ 5% a ≤ 10%)	36 (16,98)	23 (10,85)	
Alta (> 10%)	34 (16,04)	14 (6,60)	
Vacina contra doença pneumocócica			0,135
Baixa (< 5%)	144 (67,92)	158 (74,53)	
Média (≥ 5% a ≤ 10%)	33 (15,57)	30 (14,15)	
Alta (> 10%)	35 (16,51)	24 (11,32)	
Vacina pentavalente e hexavalente			0,502
Baixa (< 5%)	136 (64,15)	129 (60,85)	
Média (≥ 5% a ≤ 10%)	29 (13,68)	39 (18,40)	
Alta (> 10%)	47 (22,17)	44 (20,75)	
Vacina contra a poliomielite			0,921
Baixa (< 5%)	128 (60,38)	129 (60,85)	
Média (≥ 5% a ≤10%)	32 (15,09)	37 (17,45)	
Alta (> 10%)	52 (24,53)	46 (21,70)	
Classificação de risco			0,039
Muito baixo		31 (14,62)	
Baixo e médio		36 (16,98)	
Alto e muito alto		145 (68,40)	

n = número de municípios; TA: taxa de abandono; HCV: homogeneidade de cobertura vacinal.
